# A qualitative study of negative sociocultural experiences of accessing primary health care services among Africans from refugee backgrounds in Australia: implications for organisational health literacy

**DOI:** 10.1186/s12875-024-02567-2

**Published:** 2024-09-04

**Authors:** Prince Peprah, Jane Lloyd, David Ajak Ajang, Mark F Harris

**Affiliations:** 1 Social Policy Research Centre, University of New South Wales, Sydney, NSW 2052 Australia; 2 Centre for Primary Health Care and Equity, University of New South Wales, Sydney, NSW 2052 Australia; 3 Independent Researcher, Perth, WA 6000 Australia

**Keywords:** Primary health care, Access, Experiences, Africans from refugee backgrounds, Australia

## Abstract

**Background:**

Primary health care is the first point of contact for patients from refugee backgrounds in the Australian health system. Sociocultural factors, including beliefs and value systems, are salient determinants of health literacy and access to primary health care services. Although African refugees in Australia have diverse sociocultural backgrounds, little is known about the influence of sociocultural factors on their experiences of accessing primary health care services. Guided by the theoretical framework of access to health care, this study examined from the perspective of African refugees how culturally and religiously conditioned, constructed and bound health beliefs, knowledge and practices influence their experiences of access to, acceptance and use of primary health care services and information in Australia.

**Methods:**

This exploratory, qualitative study involved 19 African refugees from nine countries living in New South Wales, Australia. Semi-structured interviews were conducted and recorded using Zoom software. The interviews were transcribed verbatim and analysed using a bottom-up thematic analytical approach for theme generation.

**Results:**

Four main themes were identified. The themes included: participants' experiences of services as inaccessible and monocultural and providing information in a culturally unsafe and insensitive manner; the impact of the clinical care environment; meeting expectations and needs; and overcoming access challenges and reclaiming power and autonomy through familiar means. The findings generally support four dimensions in the access to health care framework, including approachability, acceptability, availability and accommodation and appropriateness.

**Conclusion:**

African refugees experience significant social and cultural challenges in accessing primary health care services. These challenges could be due to a lack of literacy on the part of health services and their providers in servicing the needs of African refugees. This is an important finding that needs to be addressed by the Australian health care system and services. Enhancing organisational health literacy through evidence-informed strategies in primary health systems and services can help reduce disparities in health access and outcomes that may be exacerbated by cultural, linguistic and religious differences.

**Supplementary Information:**

The online version contains supplementary material available at 10.1186/s12875-024-02567-2.

## Background

The recent large flow of people from refugee backgrounds has made refugee health a critical global public health issue [1], particularly regarding how to adequately respond to their complex and multiple health needs [2, 3]. Refugee populations, particularly those from African countries have significant health needs because of their prolonged exposure to unsanitary conditions in camps (owing to overcrowding, poor sanitation systems, lack of clean water and minimal ways to cook and store food) [4–6], traumatic and psychological experiences [7–9] and insufficient access to appropriate health care services [10–13]. Inadequate access to appropriate health services has been identified as one of the many concurrent post-migration factors causing progressive deterioration in the health of people from refugee backgrounds in Australia and many destination countries [14–17]. Thus, promoting timely access to appropriate health services is critical for overall health and well-being.

Health literacy, which is a shared function of social and individual level factors and occurs from the interaction of individual capabilities and systems’ demands and expectations is critical to health care access [18]. Health literacy, therefore, emerges from the alignment of health system and service demands with the knowledge and practices of individuals such as providing health information and services that are culturally sensitive and appropriate to the needs of people for meaningful health actions [19–21]. In this sense, countries of resettlement are responsible for promoting health systems and services that are health literacy responsive by implementing strategies and interventions to make it easier for all patients, including those from refugee backgrounds to navigate systems, understand health information, engage in the health care process, access appropriate and equitable services and manage their health [22–24]. Health systems and services that are created and built by the dominant culture and for the mainstream population can often remain inaccessible to many marginalised and culturally and linguistically diverse groups such as refugees due to their complexities and monocultural approaches to care delivery [25, 26].

Recent studies have, therefore, documented some key factors that form critical organisational health literacy components in promoting health care service accessibility and appropriateness [23, 24, 27]. These include enhancing communication with health users and their carers/families, promoting access to and navigation in the health care systems and services, fostering patient active engagement in the care process, instituting a multidisciplinary workforce and staff with health literacy-related knowledge and skills, establishing organisational culture and systems that support health literacy and meeting health users’ needs, including the provision of language support services such as interpreters and plain language services [27].

For instance, in Australia, primary health services and professionals have access to language services such as free phone and on-site translation and interpretation services through the Translating and Interpreting Services (TIS National) to aid communication with patients with inadequate English language proficiency, including people from refugee backgrounds [28–30]. Also, people who arrived or formally accepted as refugees in Australia are immediatly granted free access to primary health care services through the Medicare ─ universal public health insurance scheme and the Pharmaceutical Benefits Scheme (PBS) [31]. The Medicare scheme either partially or fully covers the cost of most primary health care services in the public and private health systems. All Australian citizens and people who are granted permanent residencies have access to fully-covered health care in public hospitals [32]. These arrangements exist to reduce barriers and promote access to health and social services for all people, including those from refugee backgrounds. However, such provisions do not imply that people from refugee backgrounds can access services [33]. There are a range of other barriers such as racism, feelings of inadequacy and lack of understanding of the Australian health care systems that limit access to services among this population [34–38].

More importantly, growing evidence suggests that among people from refugee backgrounds, sociocultural factors, defined as a broad range of societal and cultural influences that shape people’s feelings, thoughts, behaviours, understanding, knowledge and health outcomes [39], are among the critical determinants health literacy and access to appropriate/optimal health services [40–42]. Some salient sociocultural factors such as religion, culture and language, are essential health determinants and have been reported to be associated with several health outcomes and disparities, including health behaviours (health access and utilisation, diet, health screenings and physical activity) [43, 44] and illness (e.g. diabetes, cancer, depression and cardiovascular disease) [45, 46]. For instance, among people from refugee backgrounds, culture, which refers to shared beliefs, norms and values held and practiced by a defined group of people, is a significant determinant of health literacy [47–49], health service use and outcomes [14, 50–53].

Cultural norms, belief systems and practices affect whether and when people use services and how information from health care services and professionals is received and interpreted [54, 55]. Evidence from Australia suggests that cultural practices such as family involvement and gender preferences in care, shape people from refugee backgrounds’ access to health services [50, 51, 56]. Cultural beliefs, including beliefs in traditional or alternative medicine influence how people from refugee backgrounds seek health care and their understanding of preventive health care [57]. Therefore, health services must meet the target group’s expectations, values and belief systems regarding health and health care if they are to be responsive. However, in-depth information on sociocultural challenges that African refugees face within the Australian health system is limited. For instance, in two Australian systematic reviews on refugees’ experiences using health care services [58, 59], only 12 of the 44 studies involved African refugees and only six focused entirely on Africans from refugee backgrounds. Although other primary research has focused on African refugees' experiences with primary health care services, these studies were restricted to access to either medicines or a particular setting or service [10, 29, 59].

Accordingly, having established in an earlier paper (Peprah P, Lloyd J, Gitau L, Harris M: Migration and health literacy: A qualitative analysis of health literacy among African refugees in Australia, forthcoming) that culture, religion and previous experience with health systems influenced health knowledge and health practices of African refugees in Australia, this study extends the evidence and arguments in the previous study. It examines how culturally and religiously conditioned, constructed and bound health beliefs, knowledge and practices practically influence access to, acceptance and utilisation of primary health services and information among this population in Australia. It also examined the implications of their experiences for the health literacy of organisations and health professionals working with this population.

This study offers meaningful information to help develop tailored and innovative intervention strategies for addressing some of the health care and health outcome disparities among this population. In addition, understanding the implications of the participants’ experiences for primary care services’ health literacy in Australia is the first step in preparing primary health care services and professionals to deliver health literacy responsive care to this population. Thus, this paper adds to advancing health care equity in primary care services for people of refugee backgrounds and particularly those who are recialised like Africans living in Australia [38, 60].

### Theoretical framework

**Fig. 1:**
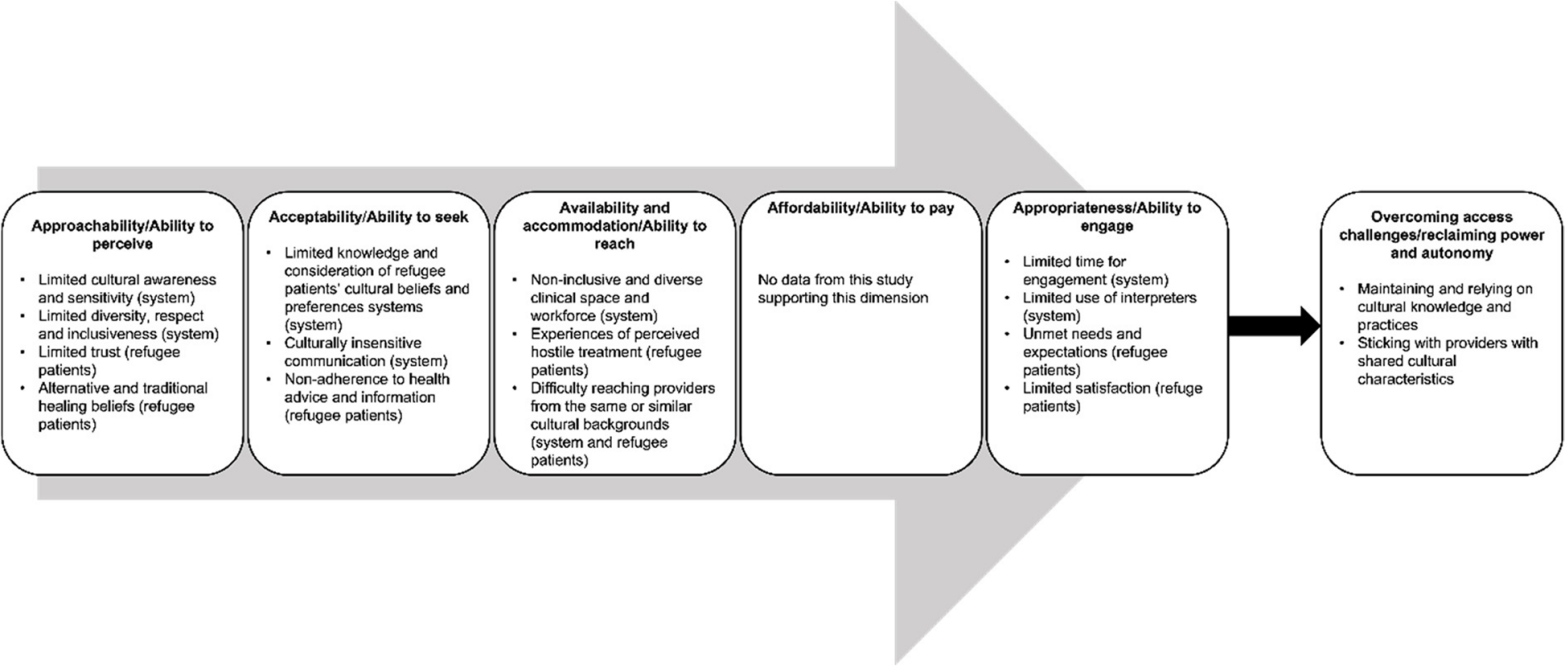
Challenges in the process of accessing primary health care as reported by the study participants. Modified from [33]. The first five boxes represent the dimensions of accessibility in Levesque’s framework. The bold arrow and the last box show new insights concerning what refugee patients do in their attempt to overcome the challenges in accessing health care and take control over their health

This study used the conceptualisation of health care access provided by Levesque and colleagues as the theoretical framework [33] (see Fig. 1). Access to health care according to this framework involves a fit between the health care system and the health of the patient, where the patient identifies health care needs, searches for health care services, accesses the services and addresses the needs [33, 61]. By contrast, health care services are expected to be acceptable, accommodative, accessible, affordable and available [33, 61]. The model incorporates the characteristics of providers/services (supply-side) and individual patients (demand-side), each identified by the five dimensions. Patients’ side dimensions include the ability to perceive, seek, reach, pay and engage with services. Provider side dimensions are approachability (denotes how users with health needs can identify and get some forms of existing services that have an impact on the health of the users), acceptability (relates to sociocultural factors that influence the likelihood for people to accept aspects of health service, e.g. cultural identity of providers), availability and accommodation (refers to the existence of services and how services including the physical space and health professionals can be reached), affordability (the financial capacity for health users to spend resources on appropriate service utilisation and appropriateness (denotes issues such as the fit between health services and health users need) [33, 62]. Hence, health care systems and services can amplify or weaken access to services such as primary health care services among vulnerable populations, including refugees [15].

## Methods

### Setting, design, and participants

This paper is based on 19 semi-structured interviews with African refugees developed from a larger interpretive, qualitative research examining health literacy and cultural responsiveness of primary health care services and professionals in Australia [63, 64]. The larger study has also conducted interviews with service providers and other key stakeholders from several primary health care organisations across three states, including New South Wales, Victoria and Queensland. This approach allowed for an in-depth exploration of participants’ experiences based on reality [65]. Specifically, this paper reports findings from questions regarding participants' negative experiences about the Australian primary health services. Notably, during the interviews, participants generally shared mixed experiences, but this paper focused on their bad experiences. The participants were from nine African countries and had stayed in camps before coming to Australia. The participants included 11 women and eight men and ranging in age from 30 to 55 years. The number of years spent in Australia ranged from four to 15 years. This study was approved by the South Western Sydney Local Health District Human Research Ethics Committee (Ethics Approval Number: 2021/ETH11161).

### Recruitment and data collection

Participants were identified through contact with the African community and organisations providing services for refugees with subsequent snowballing [66, 67]. All the initial participants enrolled in the project were asked to invite others who met the study criteria. They were eligible to participate in the study if they were originally from Africa, lived in South Western Sydney, spoke and read English, were 18 years and above and could provide written informed consent. All the participants invited through snowballing were screened to ensure that they were eligible to participate. Participants were recruited considering maximum variation and balance in the sample, as they were consciously selected [68].

Semi-structured interview guide was developed and refined by the researchers through literature review, reflective meetings and feedback from pilot interviews (see supplementary file 1). Interviews were conducted by the first author [PP], who had experience in qualitative interviews and shared an African background, but did not come to Australia as a refugee and was unfamiliar with any of the participants before the study. The interviews were conducted in English, using an institutional Zoom platform. The interviews lasted approximately 35 min (30–60 min) and were audio recorded. All participants provided informed consent (verbal or written) after reading the participant information sheets and consent forms. Participants were offered a $30 gift voucher as a token of appreciation for their time after completing the interview. Audio recordings were transcribed verbatim and cross-checked using the original audios. The information power from the data was deemed sufficient according to the use of established theory, quality of the conversation and the analysis strategy [69].

### Data analysis

As an exploratory study, thematic analysis guided coding and analysis using inductive and bottom-up approaches to theme generation [70, 71]. Thematic analysis requires thick data to create adequate information power [70]. Themes generated from the data do not represent domain summaries but reflect participants’ experiences and patterns of shared meaning centred around a dominant meaning-based concept [70, 72]. Specifically, transcripts were reflectively and reflexively read through multiple times for data familiarisation and deeper understanding beyond their semantic meaning [70]. Transcripts were iteratively coded into inductive codes and categories were developed to structure and organise the data. These codes and categories were checked across the dataset, and additional codes were included when identified. The themes were iteratively developed by searching, identifying, merging and refining codes and categories. Access to the health care theoretical framework [33] was used as an analytical lens throughout the analysis. Nvivo software (version 12) was used for data management.

## Results

Four common and interrelated themes were identified across participants’ experiences. The participants’ experiences captured in the first theme showed that cultural belief systems, knowledge and preferences were not understood and were not given the expected consideration by service. In addition, services provided information and advice to refugee patients in ways that caused undesirable outcomes such as feelings of shame. The second theme demonstrated that the clinical environment, including the workforce and physical space, was not inclusive or diverse, leading to refugee patients’ sense of being unwelcome in health care settings. As a result (theme three), participants experienced significant unmet needs and expectations regarding services. In response (theme four), participants resorted to familiar means, based on pre-existing cultural knowledge and traditional healing practices, to exercise their agency in health care decisions and behaviours.

The findings generally support four dimensions in the access to health care framework, including approachability, acceptability, availability and accommodation and appropriateness, as represented in Fig. 1. A description of these themes is provided below. Quotations are assigned to prefixes RP (Refugee Participant), followed by the participant number.

### Experiences of inaccessible, mono-cultural, culturally insensitive and unsafe information-giving approaches

This theme captures participants’ experiences that mainstream services did not recognise and account for their beliefs and practices in care delivery.

Participants expected services and providers to obtain and assess detailed information about their cultural group background, membership and identity and to gather deeper insight into their health belief systems. However, the data demonstrated that the participants’ experiences were that services and providers did not check and understand their cultural backgrounds and identities. Many participants reported that some health providers made incorrect assumptions and generalisations about their cultural backgrounds, identities and needs; asked culturally insensitive questions; caused distress by reminding them of their past; and probed sensitive topics in a culturally insensitive and inappropriate manner.

*… there are certain questions… that they ask…* [that seek to group] *… Africans together.* [Questions like] *… what type of FGM [Female Genital Mutilation] was performed on you? Instead of asking me, have you done FGM… it’s like when you go there, as soon as they see you’re black, they think you’ve done it… [RP 1]*

The above quotation implies that providers engaged with participants based on assumptions and inherent generalisations. The experience of perceived lack of cross-cultural exploration and culturally insensitive communication by providers further emerged. Some participants mentioned that some health care providers laughed at them to believe that evil forces can impact one’s health and that the only way to remedy the situation is through spiritual means.

*…* [a] *person can be possessed by an evil spirit… I was talking to these people [referring to providers]* [and they] *laughed…* [at] *me… It’s good, you can laugh…* [at] *me… the bible… recognises that there are evil spirits. So, the evil spirits can also work in a person which can manifest the signs of mental issues. But… a good priest* [or]* pastor… can chase the evil spirit and the man can be cured. [RP 19]*

In line with the above, many participants believed in complementary and alternative medicine such as herbal medicines, for treating health conditions and maintaining health. However, these participants lamented that health care providers only accepted and prescribed Western medications.

*… the GP know*[s] *only tablet… the tablet…* [makes my condition gets] *worse… maybe…* [the tablet is made for the whites].* [RP 8]*

Some participants further stated discussions with their providers about their interest and belief in traditional medicines, as well as that Western drugs worsen their health conditions. However, their experience was that their providers ignored such preferences and interests. One participant who needed traditional medicine and discussed it with a provider narrated how the provider reacted:

*…he [referring to the provider] just said I don’t need it when I said there are medicines from Africa which will help me. [RP, 19]*

The above experiences of the participants indicate that providers with a better understanding and awareness of refugee patients would engage with them to understand their preferences. For instance, providers would first explore and listen to patients’ beliefs about the causes of illness, health conditions and medication and what is important to them to negotiate safe and effective options. In addition, instead of laughing about participants’ belief systems around disease aetiology and ignoring or neglecting preferences regarding medications, good health literacy of the providers would mean engaging with patients to reach an understanding that, for instance, it is safe to see a spiritual healer however, these drugs need to be considered.

It was clear from the interviews that adequately responding to the knowledge and practices of refugees required clear, effective and culturally sensitive communication and strategies that instil trust in the services and information given. These included giving patients health information/messages more respectfully to avoid stigma and patients’ feelings of shame and being restricted from practicing their cultural beliefs. It was the participants’ experience that services gave them instructions and orders that they felt were against their cultural beliefs, knowledge and health practices. For instance, some female participants narrated how providers stopped them from showering their newborns immediately after giving birth.

*… they say, no, you cannot wash the child or give them a bath until after 24 hours. From where I’m coming from …. We link…* [bathing]* …* [to] *body odour… that if you…* [are] *not [given a] bath within the first 24 hours you [will] have body odour for the rest of your life… that’s…* [our] *cultural belief. I want my kids to be given a bath immediately after they’re born…* [but]* the nurses… are against it. [RP 1]*

One participant also recalled her feelings of shame about how a GP reacted to her cultural practice.

[Concerning] *FGM… I remember one time I went to the hospital…* [my GP noticed] *I have done it [and] he was scared…* [which] *… freaked me out…* [He was] *oh, what is this? Are you okay? That was bad* [they did this to you]*. But from my culture, that is what we do… but he made me feel… uncomfortable…* [he was]* trying to refer me to a gynaecologist to make sure I was okay. Well, of course, I was okay. [RP 5]*

The above experiences suggest that communication from services was culturally insensitive. These experiences demonstrate health services and/or providers may lack the knowledge in delivering culturally sensitive information. Participants said that they did not follow the advice given in such a way that it was not trusted, as exemplified below.

*… they told me not to bath my child, but… after… they left, I did. I asked my husband to help me, and then my mum came in and supported me as well. [RP 5]*

Suppose the providers had communicated the same information with a higher degree of health literacy and cultural sensitivity, the participant may have accepted and followed the instruction.

### Impact of the clinical care environment

This theme is related to the view that people from different cultures live in Australia. As a result, the health services environment such as hospitals, clinics, and medical centres, should be culturally inclusive and welcoming:

*There are people from all different cultures living in this country. I think more effort could be made to make sure people feel welcome and that we take the time to understand different needs. So, we need to create spaces that people can walk into and feel it is the place for them to go and get their health care. [RP 3]*

The above views of the participants suggest that health services with a full array of health care providers from different cultural backgrounds can reinforce effective, safe and culturally sensitive interactions between refugee patients and professionals. However, the participants saw themselves in an unfamiliar environment because of the perceived lack of multicultural staff. They expressed concerns and frustrations about their inability to find providers from the same or similar cultural backgrounds. They felt that people from African countries were often not recruited into the medical field in Australia.

*… I think sometimes it’s a bit hard trying to find a GP that understands… understands your cultural beliefs and your perspectives in terms of health care as well… It’s so hard… to find a black doctor… even in Sydney… there…* [are] *Indians around which can be like our… skin* [colour]*, but to actually find services that have black doctors, it is so hard. [RP 18]*

The participants felt safe, comfortable and empowered in an environment with professionals who shared their cultural characteristics.

[When you find] *… a black person…* [they] *… can relate to me… understand me…* [and my] *… issues that I go through… when you do find a doctor…* [from] *other nationalities or… race… they’re not culturally sensitive, they don’t understand our issues or our plight that we go through… obviously when I’m talking to you, you can understand more some of the problems and challenges that I’m talking about…* [when you find a black doctor]* you can have that connection straight away. [RP 18]*

The preceding quotation suggests that participants engaging with health professionals from the same cultural background may have received care that met their expectations because of the shared understanding between them and the providers. They may also have a better understanding of their health because of rapport, effective engagement, knowledge and trust.

Culturally diverse patients such as the study sample can face significant challenges in finding their way to and around clinical care facilities when such facilities are not designed in a way that is easy to navigate, such as having culturally appropriate signage or pictograms. They may also find it challenging to feel they belong when there is an absence of cultural artwork they can identify with. Meanwhile, experiences shared by the participants indicated that the physical environment of health care settings such as clinics, also created an unfamiliar environment. The participants maintained that many Australian clinical care environments lacked cultural representations such as artworks, paints, buildings and other objects, to promote easy navigation and a sense of belonging. It was revealed that appropriate cultural representations indicate welcoming and accommodating services. Some participants shared experiences of what good cultural representation felt like.

*… I know […] hospital is very good, but there are other hospitals that are not good. Even in […] Hospital now you see in the maternity ward area they have some African artifacts, which is good… If you’re looking up the ceiling* [it], it* seems like something you can identify with. It really makes it good. [RP 1]*

The above narrative can encourage return visits by the participants because of their perceived positive experience with the clinical environment. Regardless of the positive experience, the data also showed negative experiences of a sense of unwelcome, unfair treatment and aggressive behaviours of health professionals in health settings. Many participants shared their perspectives on how some providers treated patients from African backgrounds. They mentioned that Africans are treated harshly by some health professionals compared with patients from other backgrounds.

*… they don’t have that patience when they’re dealing with Africans… they’re not sensitive to the needs of Africans… there is that passive aggressive racism in there. I’ve seen that a lot… in health care. That passive aggressive way of dealing with Africans… the way the nurses talk to a patient that is not an African* [is different] *… they are super caring… they babysit them… but…* [for an]* African person, they just throw things at you… [RP 7]*

Some participants shared evidence of service disengagement owing to the experience of health professionals’ perceived discriminatory and unwelcoming attitudes.

*… that’s why you see a lot of Africans… are* [withdrawing from seeking] *medical support* [and] *help… because they don’t want to go back to the hospital…* [for] *someone* [to]* traumatise them. [RP 7]*

The above quotation indicates that participants’ experiences of hostile treatment may serve as a barrier to provider-patient communication and service engagement and access on the part of the participants.

### Meeting expectations and needs

This theme describes the participants’ expectations and unmet needs for health services and providers. The participants had grown up in African nations, stayed in camps and were accustomed to certain care delivery services and models. As such, participants expected to access the same or even better services in Australia, however, their expectations were not met.

The participants mentioned a lack of respect, quality engagement and trust as issues associated with services. African refugees expected services and providers to engage with them on topics other than medical matters to build trust for effective communication and engagement to promote their health knowledge. However, the conversations were medically focused.

[There] *are services… available to you… but they don’t have that time to create a rapport. Some people go there, not only to try to seek medical help, but to understand certain things going on in their life… I* [have] *found* [that] *GPs… don’t probe their patient… they don’t spend time to get to know them… they always try to push them out of the door. [RP 7]*

The above participants' perspectives indicate that professionals working with them may not be able to know and understand their overall well-being and other determinants of their health because of the perceived lack of quality engagement. For instance, some participants expected providers to at least know and call them by their names to initiate conversations after visiting them for a long time. However, they revealed that providers often failed to call them by their names, indicating a perceived lack of respect. According to the participants, this experience serves as a barrier to rapport trust-building and effective provider-patient engagement.

*… some of them… don’t even take the time to learn* [even] *your name… like this is a place that you’ve been going there for years, and they don’t even bother just to even know your name or know more about you.* [Anytime you go there] *… they have to ask… your name again… I’m like I’ve been coming here for years now, you should know my name… simple things like that go a long way… [RP 18]*

Effective communication and engagement between providers and linguistically diverse patients such as the study sample, requires access to culturally appropriate language services such as trained interpreters. Although some participants needed interpreters to communicate with health professionals, they did not use them. Some participants indicated that providers mentioned that they did not use interpreters because of short consultation time, limited experience of providers in interpreter use, trust and privacy issues. Consequently, many providers were sometimes reluctant to see people from non-English-speaking backgrounds.

*Most of these doctors… don’t like using interpreters, so therefore they are providing you with a service based on assumption… there are… people who will go to doctors…* [with language issues, but the doctors] *… don’t want to use interpreters, and that is not good. [RP 1]*

This experience can compromise effective communication and engagement between providers and patients who cannot speak English.

Lack of family involvement in care was another subject of unmet or mismatched expectations. Many participants mentioned that their family members were often excluded from clinical care. Some participants echoed their frustration with the lack of family support in clinical care.

[Someone in our community was having a rash and was isolated]*. We as Africans, if we are sick… we need someone by our side to force us to* [even] *eat…* [we do not isolate sick people] *… our culture doesn’t demand that… our culture, no matter* [the sickness] *… even if you have Ebola, we can still…* [come]* close to you. [RP 10]*

It is clear from the preceding narrative that in participants’ cultures, decisions and decision-making regarding health may often occur as a collective and community activity of family, relatives and peer groups. Thus, the experience shared by the participants implies that services should involve families and relatives in care to enhance people’s ability and willingness to engage with relevant information, messages, resources and support for them to make their health decisions effectively.

### Overcoming access challenges and reclaiming power and autonomy through familiar means

This theme captures participants’ actions and strategies to overcome the challenges they face in accessing services and reclaiming power and autonomy over their health and well-being.

Interviews revealed that many participants resorted to familiar ways because of the various sociocultural challenges in accessing the services. Participants used these strategies to overcome difficulties, reclaim power and autonomy, exercise their agency in health care decisions and behaviours and improve their health. The use of traditional healing practices, reliance on cultural knowledge and sticking with providers with shared cultural characteristics were familiar avenues to which participants often resorted.

Traditional medicines were a means for the  to take control of their health, since they reported that providers did not accept or encourage conversations about the use of herbal-based medicines. Participants expressed trust in the efficacy of traditional medicines such as herbs. The participants’ experience was that conventional medicines such as herbal products were compatible with their cultural belief systems, effective, and not associated with any perceived adverse side effects.

*… I think that we are in a system…* [that we cannot change it]*. But if we are clever…* [and] *intelligent, we have to… access… what we know…* [it helps us as Africans] *… for example,* [if you know] *that that tree… those leaves [and] that plant can help you to cure* [your disease, you should]* use it. That is not a problem. [RP 19]*

Another participant was frustrated about the use of medicines such as Panadol and, as a result, resorted to using traditional healing practices.

[If you are not feeling well], *they tell you* [to]* take Panadol… but you are not going to be happy with it… you use your own way to serve yourself. [RP 8]*

The above quotes indicate that participants seek medicines that are concordant with their cultural beliefs and practices. Access to such approaches may promote a sense of empowerment and autonomy over health. This suggests that providers should encourage conversations about beliefs and practices regarding prescriptions/medications with patients from culturally diverse backgrounds such as the study sample.

The use of cultural knowledge to reclaim power and autonomy was evident throughout the participant interviews. For instance, female participants in this study maintained and practiced their cultural practices such as giving newborns bath immediately after delivery.

*…we usually give them* [referring to babies] *water as well,* [but] *they’re against it as well,* [but]*… we do* [it]* in our community… immediately within two hours, we give them… give them water. [RP 1]*

Though the participants’ cultural practice and knowledge of giving newborn babies water appears to be at odds with the providers’ advice, they are still interested in doing so because of their quest to exert control and authority over their health decisions and health practices. This quote also implies that the participants also wanted to maintain their cultural practices.

Overcoming language challenges by adhering to providers who speak their own language was typical.

*. … since I come here… in 2000… I have been seeing one GP until now….* [because he]* understands my language and I can understand his language as well… [RP 11]*

Seeking care from service providers who speak their native languages can promote their interaction with providers, improve their health knowledge and promote empowerment.

## Discussion

This qualitative study has unpacked how African refugees' unique culturally and religiously conditioned, constructed and bound health beliefs, knowledge and practices influence their experiences of access to, acceptance and use of primary health care health services and information in Australia. It also discusses the implications of these experiences for organisational and practitioner health literacy. We observed from the findings that while some of the participants have stayed in Australia for many years, their experiences regarding primary health services were not different from those who have lived in the country for a few years. The findings are discussed under four broad themes, including participants’ experiences of services as inaccessible and monocultural and provided information in a culturally unsafe and insensitive manner, the impact of the clinical care environment, meeting expectations and needs, overcoming access challenges and reclaiming power and autonomy through familiar means. The themes support all the dimensions of the access to health care services framework except the affordability/ability to pay dimension. This observation could be due to the existing health insurance coverage arrangement in Australia that all people who have been formally accepted as refugees are given Medicare, which lessens financial barriers to most primary health care services [31].

Participants shared that providers did not explore issues and beliefs that were important to them such as alternative and traditional healing approaches including spiritual healing and herbal medicines. Participants believed in care that responded to and supported their physical, emotional, social and spiritual needs; however, it was their experience that providers ignored their preferences. To some extent, this experience deterred participants from seeking conventional care, which follows the second dimension in the access to health care framework of acceptability of services and health users' ability to seek services. The experiences of the participants highlight the influence of social and cultural factors on the possibility of health users accepting health services as well as the judged appropriateness for the user to obtain care [33]. The participants' experiences are connected with the requirement for health services and providers to be health-literate [24, 73, 74]. Organisational health literacy requires services and providers to understand the cultural backgrounds, identities and beliefs of patients [47]. It also demands that services adopt culturally safe and cross-cultural methods such as Kleinman's explanatory model [75] to explore, gather and assess patients' cultural identity and belief systems and preferences to negotiate with them in reaching mutually desirable health decisions and outcomes such as changing the health behaviours of patients [47, 76, 77].

Similarly, responding to the health knowledge, beliefs and practices of refugee patients requires clear and culturally appropriate communication skills and strategies from health services and providers to promote trust in information and services [23, 78, 79]. However, participants were asked questions and providers reacted to their practices in ways that stigmatised and caused them to feel ashamed. There were also participants' experiences of information and advice, which could be best health care practices not being communicated clearly and in a way that respects their cultural norms and practices. Previous studies involving people from refugee backgrounds reported similar experiences [40, 58, 80, 81]. The participants’ experiences generally demonstrated situations in which health instructions based on best practices were poorly and insensitively communicated. This leads to compromised communication due to a lack of recognition of cultural needs and beliefs. Compromised communication and engagement may also lead to poor health literacy among the participants, as effective communication enhances health literacy [64]. This finding also aligns with the approachability and ability to perceive dimensions within Levesque et al.'s access to the health care framework, highlighting how prior experiences shape one's perceptions and experiences about the health care system and services [33].

Therefore, this finding reminds services and providers that it is their responsibility to communicate health advice and instructions considering patients' beliefs and practices even when such information does not conform to their cultural needs. This can be done by services and providers who understand that an individual's concept and understanding of health may differ, affecting how people receive, process and accept (or reject) health information [47]. Through this understanding, providers and patients reach a desirable consensus regarding the right course of action. Failure to do so can result in undesirable outcomes such as patients disregarding advice and stopping access to the needed health care services, as they are not attuned to their cultural needs.

The organisation of services also determines health care access and utilisation regarding how the physical space and health professionals within the services can be reached, represented by the availability and accommodation dimensions in the access to health care framework [33]. In support of these dimensions in the access to health care framework, participants perceived the clinical care environment and workforce as non-inclusive because they found it difficult to find providers from culturally diverse backgrounds and cultural representations such as artworks and culturally appropriate signage and pictograms that promote navigation. Participants, therefore, saw themselves in an unfamiliar and unwelcome environment, which acted as a deterrent to access and receive care. This finding supports many studies that commented on the "Whiteness" of the Australian health system and services, reflecting a lack of inclusiveness and diversity [82–86]. For instance, in a multi-method qualitative study among organisations (including primary health care organisations) working with people of refugee backgrounds in Australia, it was found that "Whiteness" operated through the workforce and how physical spaces are constructed [85]. Similar to the present study's findings, the study further reported that most participants felt discomfort in these contexts [85]. Experiences of seeking care in an unfamiliar environment reinforce the importance of services ensuring diversity and inclusiveness in both the workforce and the physical space, as highlighted in various organisational health literacy frameworks [24, 77, 87–89]. Such diversity and inclusiveness can promote greater navigation and access, engagement and empowerment owing to positive experiences with services.

Unmet expectations and needs emerged based on the participants' migration experiences and sociocultural norms that shape expectations of Australian health care services. Unmet expectations and needs were related to culturally insensitive services, lack of engagement, family support and linguistic issues. It was the participants' experience that services did not use interpreters to foster effective communication and engagement, which aligns with the previous findings [10, 29, 90]. For instance, participants mentioned cases in which providers did not accept patients from refugee backgrounds because they lacked English proficiency skills and had limited consultation time to use interpreters. Lack of engagement affects trust building and effective communication, resulting in mistrust or distrust, lower health care use and poorer health outcomes [52, 91]. In addition, instances in which health care providers did not accept new patients based on language ability suggest that other institutionalised forms of discrimination exist within services. Experiencing communication issues due to the lack of interpreter use by health service providers may increase distrust and reinforce negative experiences, which can render health care services inappropriate [15]. This is in line with the final dimension of access to the health care framework, appropriateness and ability to engage [33].

This attitude may lead to marginalisation and poor health outcomes as professionals avoid time-consuming patients [92]. This finding confirms the pervasive monolingual mindset of many health care providers in Australia [93]. Thus, this study recommends policies and strategies that promote quality engagement, good communication, humane treatment and trusting relationships between health care providers and refugee patients to increase trust and positive experiences [94]. This recommendation is within the framework of organisational health literacy, requiring all patients, irrespective of their cultural and linguistic backgrounds, to access culturally appropriate language services such as interpreters [95].

In the face of perceived sociocultural challenges and unmet expectations and needs, participants adopted familiar strategies and practices to overcome difficulties, reclaim power and autonomy and exercise their agency over their health. This finding to some extent aligns with the ability to perceive, seek and engage dimensions in the access to the health care framework [33]. Participants mostly maintained traditional practices such as using traditional healing practices, relying on their own cultural knowledge and sticking to providers who spoke their language. These practices have been reported in previous studies [17, 57, 96–98]. It is important to emphasise that, at times, participants may successfully take control of their health through these means. For instance, finding a provider who speaks their native language may promote effective communication and engagement, leading to greater information acquisition and understanding and empowering them to make decisions. However, maintaining traditional practices, including giving newborns water immediately after birth, which seems to be at odds with evidence-based health care practices and medical advice, might not lead to a good long-term outcome [99]. These practices, therefore, serve as a hint for services to respond appropriately to African refugees' long-held belief systems, practices and knowledge, with a higher degree of health literacy. These findings also suggest that health professionals should engage with refugee patients to explore and understand their beliefs.

### Practical implications for system and services’ health literacy

The findings from this research are relevant for understanding the impact of sociocultural factors on the access and use of health care services in Australia among African refugees. The findings further build on the access to the health care framework. The participants' experiences also highlight the importance of services and providers being health-literacy-responsive. The findings reveal an essential factor that needs to be acknowledged by health policymakers and services, especially those providing care for people from refugee backgrounds. It should be acknowledged that people from refugee backgrounds are not homogenous groups. As a result, there are sociocultural differences in this population that lead to diverse health needs and knowledge. These sociocultural differences and experiences significantly influence various aspects of refugees' lives, including health knowledge; therefore, they all need to be integrated into health services and providers' conceptualisations of clinical practice. Providers cannot care for patients without dealing with the sociocultural topics discussed in this study, because patients routinely bring these issues to the clinical environment and into treatment. Health services and professionals, therefore, need to improve their literacy about their patients’ needs, expectations and preferences. This requires health services and professionals to adopt patient/person-centred care approach to help them orient services and medical practices towards patients [100–102]. Yet, if this person-centredness is to be effective, the implementation of cultural safety and humility and trauma and violence informed approaches and policies across primary care services has to be taken seriously. Such approaches and policies would make health care services more sensitive and responsive to the needs and expectations of patients with varying health beliefs, knowledge and practices. Failure to do this would make the services less appropriate and relevant to refugees  outside the traditional target groups.

By doing the above, health services and professionals would accept that there is a need to embed cultural and health literacy responsiveness in their core systems, policies and practices to reduce health access and outcome disparities, which may be exacerbated by cultural, language and religious differences. Evidence-informed policies and strategies that can enhance organisational cultural and health literacy include designing health care settings to be welcoming to all, capitalising on avenues to integrate health literacy and cultural plans and interventions and co-designing and providing consolidated health literacy, cultural and linguistic training for health care professionals and other staff [47]. Other strategies could be employing inclusive and multidisciplinary staff such as cultural brokers and community health workers, health educators, interpreters, navigators and adapting systems to capture culture, language and health literacy information/data to inform quality improvement activities [47, 64].

### Strengths and limitations

This study involved participants from different cultural backgrounds to ensure sample diversity. For instance, 19 Africans from refugee backgrounds from nine African countries were included in the study. Again, this study did not use interpreters during the interviews, promoting direct communication and conversation between the interviewer and participants. Direct communication is likely to positively impact data reliability and minimise information lost in translation. Despite these strengths, this study has some limitations that need to be addressed. The study recruited Africans from refugee backgrounds from different countries, who might have had different experiences accessing primary health care services in Australia. For example, in some African nations, English is either the official language or one of the official languages; hence, such participants may experience fewer linguistic barriers. In addition, some African countries have intercultural health systems; thus, participants from such countries may face severe challenges with Australia's dominant Western model of care. However, this study did not evaluate differences in participants' experiences according to refugees' country of origin. The study also focused on refugees’ experiences with primary health care services. Thus, future qualitative studies can explore differences in experiences based on countries of origin and service users' accounts to inform more tailored access interventions.

## Conclusion

This qualitative study offers insights into the experiences of sociocultural factors that are important to African refugees when accessing health care. It also demonstrates the implications of these experiences for organisational and provider health literacy in responding to these refugee patients. These findings can be helpful to primary care organisations and providers working with this group when enhancing their health literacy to break down sociocultural barriers to health services, decrease health disparities and improve quality and culturally responsive care. The results further provide direction for future targeted and tailored initiatives and solutions to barriers to primary health care services among African refugees. The findings suggest organisations and professionals should adopt integrated strategies and approaches such as co-designing and integrating health literacy, culture and linguistic interventions and strategies and use culturally sensitive, safe and humble communication and engagement methods. The data also suggests a need for services to address the systemic issues of culture and add impacts of racism and discrimination of refugees, particularly Africans in Australia as a contextual underpinning of health care and primary care specifically.

## Supplementary Information

Supplementary Material 1.

## Data Availability

All datasets used and/or analysed during the current study are available upon request. Please contact the corresponding author [Prince Peprah] if you want to request the data from this study.

## Abbreviation

GP: General Practitioner
